# Supplementary health benefits of soy aglycons of isoflavone by improvement of serum biochemical attributes, enhancement of liver antioxidative capacities and protection of vaginal epithelium of ovariectomized rats

**DOI:** 10.1186/1743-7075-6-15

**Published:** 2009-04-09

**Authors:** Tu-Fa Lien, Yu-Lin Hsu, Dan-Yuan Lo, Robin YY Chiou

**Affiliations:** 1 Department of Animal Science, National Chiayi University, Chiayi, Taiwan; 2 Department of Veterinary Medicine, National Chiayi University, Chiayi, Taiwan; 3 Department of Food Science, National Chiayi University, Chiayi, Taiwan

## Abstract

**Background:**

In the literature, supplement of soy aglycons of isoflavone as estrogen agonists in improvement of serum biochemical attributes, liver antioxidative capacities and vaginal epithelium protection has been meagerly investigated. In this study, ovariectomized (OVX) rats were used as an animal model to simulate post-menopausal status. Supplementary health benefits of soy aglycons of isoflavone (SAI) on improvement of growth and serum biochemical attributes, enhancement of liver antioxidation-related capacities and protection of vaginal epithelium of the OVX rats were assessed.

**Methods:**

As an *in vivo *study, 30 OVX Sprague-Dawley rats were distributed into OVX (positive control), OVX/LSAI (low SAI group – supplemented with 0.0135% SAI being equivalent to 80 mg per day for a 60 Kg-human), and OVX/HSAI (high SAI group – supplemented with 0.027% SAI) and 10 rats with sham operation as negative control fed with basal diet.

**Results:**

The average daily gain (ADG), feed intake and feed/gain ratio were higher for the OVX groups than the sham group (*P *< 0.05). Serum isoflavone concentrations of the OVX rats were increased by SAI supplementation. In comparison, significantly lower serum cholesterol and LDL (low-density lipoprotein) levels, and higher HDL (high-density lipoprotein) levels were detected for the rats of OVX/HSAI group (*P *< 0.05). SAI supplementation also increased iron chelating ability and decreased values of TBARS (thiobarbituric acid-reactive substance) (*P *< 0.05) of liver extracts. Liver catalase activity and total antioxidative activity (trolox equivalency) were enhanced by HSAI supplementation (*P *< 0.05). Decrease of vagina epithelial cellular linings of the OVX rats were noticeably improved by dietary supplementation with SAI.

**Conclusion:**

Diets supplemented with soy aglycons of isoflavone have conferred health benefits to the OVX rats, in comparison to the sham rats fed with basal diet, by detection of higher serum isoflavone concentrations, significantly lower contents of serum cholesterol and LDL, and higher contents of serum HDL, increased iron chelating ability, lower contents of TBARS (thiobarbituric acid-reactive substance) and enhanced catalase and total antioxidative (as trolox equivalency) activities of the liver extracts, and protection of the epithelial cellular linings of vagina in the former rather than in the latter. This evidences that estrogen-agonist chemoprevention of menopausal-related cardiovascular diseases, decreased liver antioxidative capacities and epithelial degeneration of vagina could be achieved by dietary supplementation with soy aglycons of isoflavone.

## Background

Coronary heart disease, hyperlipidemia and osteoporosis are common post-menopausal syndromes which are closely related to deficiency of estrogen. HRT (hormone replacement treatment) is one of the alternative treatments in relief of post-menopausal symptoms. However, the likely tendency to induce uterus and breast cancers due to side effects of HRT is concerned. Phytoestrogens, such as soy isoflavones of estrogen agonists, have weak estrogenic activities. Soy isoflavones as alternatives of HRT to solve post-menopausal problems and avoid the side effects have been demonstrated [1]. For food safety consideration, McClain et al. [2] reported that genistein, orally administrated with mice and rats up to 2000 mg/kg, was not mutagenic or clastogenic. For Chinese and Japanese, their dietary items contain large amounts of soybean products. As estimated, they consume approximately 40–80 mg of soybean isoflavones daily. In comparison, hyperlipidemia, coronary hearty disease and post-menopausal syndromes for these Asians are markedly lower than the Europeans and Americans who consume much lower amounts of soybean products in their normal diets [3,4].

Soy isoflavones are most available and affordable source of phytoestrogen in the world. Isoflavones are polyphenols, featuring with 1–3 hydroxyl (^-^OH) groups. These ^-^OH groups are reactive, as is vitamin E, to attach free radicals in production of stable radicals and performance of antioxidants [5]. Soy isoflavones exhibit inhibitory effects on lipid and LDL oxidation [6,7] and reduction of atherosclerosis, coronary hearty disease and cancers incidence [8]. Soy isoflavones also increase cholesterol metabolic rate and reduce hyperlipidemia crisis [9]. Among the molecules of soy isoflavones, aglycon forms of genistein and daidzein are absorbed faster by human and in higher amounts than their glucosides [10]. Genistein and daidzein exert more potent estrogen agonist and antagonist activities than do other test molecules, mainly depending on level of supplementation [8]. In the literature, supplement of soy aglycon isoflavones to render estrogenic functions on improvement of serum biochemical attributes, antioxidative capacities and vaginal epithelium protection has been meagerly studied. In this study, ovariectomized rats were used as an animal model to simulate post-menopausal status. Dietary supplementation of aglycons of soy isoflavones in affecting serum biochemical attributes, lipoprotein profile, antioxidative capacities and histopathological examination of the vaginal epithelium were investigated.

## Methods

### Ovariectomy of Sprague-Dawley rats

Ovariectomy was performed when the rats female were sexually mature (ca. 11 week-old). The abdomen area of each rat was sterilized with 75% ethanol solution and opened by surgery. After the ovary was ligated and cut out, the uterus and adipose tissue were put back and sewn up. The sham rats were just subjected to opening of the abdomen and then sewn up. Rats were recovered for 2 months prior to the experiment.

### Animal treatment

Thirty ovariectomized Sprague-Dawley rats (average body weight was 226.5 ± 5.6 g) were randomly distributed into 3 groups, namely, a positive control (OVX) and two groups with dietary supplementation of soy aglycons of isoflavone (SAI). Another group of 10 sham rats was assigned as negative control. Rats of the positive and negative control groups were fed with basal diet (Table 1). Two treatments, i.e., a low supplementation group (OVX/LSAI, supplemented with 0.0135% SAI) and a high supplementation group (OVX/HSAI, supplemented with 0.027% SAI). The SAI contains 4.5% daidzein, 14.5% genistein, ca. 1.0% of other isoflavones and 80% soybean flour (Glory Biotech Co., Chiayi, Taiwan). The low supplementation level (LSAI) was estimated by an equivalency of 80 mg per day destined for a 60 kg human [11] [(80 mg/60 kg) × 6.25 (a conversion factor of adult human to rat) = 8.33 mg/kg BW (dose for rats); 8.33 mg/kg × 0.3 kg (BW of rats) = 2.5 mg/d; 2.5 mg/0.0185 kg (average daily feed intake) = 0.0135% (dietary level)]. The required amounts of SAI were deposited and mixed into basal diet. The rats were housed in individual cages and raised for 3 months. Water and feeds were free access. Room temperature and light/dark cycle was set at 25°C and 12 h, respectively. Animals used in this experiment were cared for under the guidelines stated in the *Guide for the Care and Use of Agricultural Animals in Agricultural Research and Teaching*. Body weights at initial and final stages and feed consumptions were determined. Handling and killing of the rats were in full accordance with the Institutional Animal Care and Use Committee (IACUC) guidelines for the care and management of laboratory animals.

**Table 1:** Composition of the experimental diets for Sprague-Dawley rats

Ingredients	Content (%)
Casein	20.0
Sucrose	50.0
Cornstarch	15.0
Fiber	5.0
Corn oil	5.0
DL-methionine	0.3
AIN-Vitamin premix^a^	1.0
AIN-Mineral premix^b^	3.5
Choline bitartrate	0.2
Calculated value:	
Crude protein, %	18.78
Metabolizable energy, kJ/g	14.85
Lysine, %	1.42
Methionine, %	0.49
Ca, %	0.85
P, %	0.67

^*a*^AIN vitamin mixture ingredient: Vitamin A, 22.01 IU/g; Vitamin D3, 4.5 IU/g; Vitamin E, 49.01 IU/g; Carotene, 4.5 ppm; Vitamin K, 0.5 ppm; Thiamin, 17.2 ppm; Riboflavine, 8.0 ppm; Niacin, 123.7 ppm; Pantothenic acid, 24.0 ppm; Folic acid, 5.9 ppm; Pyridoxine, 6.0 ppm; Biotin, 0.2 ppm; B_12_, 22.0 mcg/kg.

^*b*^AIN mineral mixture ingredient: CaHPO_4_, 50%; NaCl, 7.4%; K_3_C_6_O_5_O_7_.H_2_O, 22%; K_2_SO_4_, 5.2%; MgO, 2.4%; MnSO_4_, 0.35%; FeSO_4_, 0.6%; ZnO, 0.16%; CuSO_4_, 0.03%; KIO_3_, 0.001%; Na_2_SeO_3_.5H_2_O, 0.001%; CrK(SO_4_)_2_. 12H_2_O, 0.055%.

### Serum, liver and vagina sampling and preparation

Rats were sacrificed after anesthesia by CO_2_. From each rat, 10 ml blood was sampled from the anterior artery. Blood samples were centrifuged at 2000 *g *for 10 min to obtain serum and stored at -20°C. Then, liver tissue samples were taken and subjected to analyses.

For liver extract preparation, aliquots of liver tissue (ca. 5 g) were deposited into tubes with 10 ml of buffer solution (0.25 M sucrose in 1 mM EDTA-2Na, pH 7.4), pre-cooled in an ice bath, and homogenized (Ystral GmbH, D-7801, Dottingen, Germany) for ca. 1 min. The homogenized solution was centrifuged at 800 *g*, 4°C for 10 min to remove debris. The supernatant was further centrifuged at 10,300 *g*, 4°C for 10 min and the upper fraction was subjected to ultra-centrifuging at 105,000 *g*, 4°C for 60 min (Beckman L8, Beckman Instruments Inc., Bensenville, IL). The final supernatant (cytosol fraction) was collected and stored at -80°C for determination of antioxidative capacities.

### Analyses of serum biochemical attributes

Serum estradiol concentrations were quantified by a commercial ELISA kit (Active Co., Webster, TX). Cholesterol level was analyzed by an enzyme kit and an autoanalyzer (Roche. Cobas, Miras, Switzerland). Lipoprotein profiles were determined by electrophoresis and intensity of the separated specific bands was quantitatively scanned by a densitometer (Helena Co., Beaumont, TX).

Serum isoflavone levels were analyzed by HPLC and PID equipped with a C18 column at 40°C with a column oven. Detection wavelength and injection volume were 254 nm and 20 μl, respectively. The mobile phase comprised 0.1% trifluoroacetic acid (A solution) and acetonitrile (B solution). Each run was initiated with a programmed mode with A solution at 1.5 ml/min for 25 min, increased to 22% B solution in 1 min, further increased to 25% B for 14 min, 50% B for 10 min, 80% B for 5 min and finalized with 80% B solution for 60 min. Authentic daidzein and genistein (Sigma Chem. Co., St. Louis, MO) were run concurrently as standards and based for estimation of total isoflavone concentrations. The detection limit was 0.1 μg/ml.

### Determinations of antioxidative capacities of liver extracts

Superoxide dismutase (SOD) activity of liver extracts was determined following the Ellerby and Bredesen [12] method. Briefly, 15 μl of the stock 6-hydroxydopamine was deposited into 1 ml of 0.05 M sodium phosphate buffer solution containing 0.01 mM diethylenetriaminepentaacetic acid (pH 7.4) and 10 μl liver extract and subjected to absorbance monitoring at 490 nm (Hitachi U-2000, Tokyo, Japan) in quantification of 6-hydroxydopamine auto-oxidation. One unit of enzyme activity was expressed as mg of protein resulted in 50% inhibition of 6-hydroxydopamine auto-oxidation per min. The determination was conducted in triplicate and a commercial SOD (Sigma Chem. Co.) was used as a standard reference.

Catalase activity of liver extract was measured following the method described by Ellerby and Bredesen [12]. Decomposition of 1.0 millimole of H_2_O_2 _per min was defined as one catalase unit. A commercial catalase (Sigma Chemical Co.) as a reference was run concurrently. Enzyme activity was expressed as unit/mg protein.

Glutathione peroxidase (GSH-Px) activity of each liver extract was determined following the method reported by Bhat et al. [13]. For each determination, 0.8 ml of substrate buffer solution containing 1 mM EDTA, 1 mM NaHCO_3_, 0.2 mM NADPH, 1 U/ml glutathione reductase, 1 mM glutathione and 100 mM KH_2_PO_4 _(pH 7.0) was mixed with 25 μl of liver extract and incubated at 25°C for 5 min. Then, 0.1 ml of H_2_O_2 _(2.5 mM) was added to initiate catalysis for 3 min prior to absorbance determination at 340 nm (Hitachi U-2000). One unit of activity was expressed as 1.0 nanomole of NADPH oxidized/mg protein/min.

Thiobarbituric acid-reactive substance (TBARS) in each liver extract was determined according to the procedure reported by Tarladgis et al. [14] and expressed by nanomole MDA/ml. Peroxide value (POV) was determined following the procedure reported by Sebranek [15]. Conjugated diene hydroperoxide contents were measured according to the method described by Osawa et al. [16]. Cellular protein concentration was determined by Lowry et al. [17] method.

Total antioxidant activity (trolox equivalent) of each liver extract was determined following the procedure reported by Erel [18]. Briefly, 200 μl of reagent A (acetate buffer solution containing 0.4 M CH_3_COONa and 0.4 M glacial acetic acid, pH 5.8) was mixed with 20 μl of reagent B (glacial acetic acid buffer solution containing 30 mM CH_3_COONa, 30 mM glacier acetic acid, and 2.86 M H_2_O_2_, pH 3.6). Then, 0.549 g ABTS [2,2'-azinobis-(3-ethylbenzothiazoline-6-sulfonic acid)] was dissolved in 100 ml of prepared solution. and further mixed with 5 μl of liver extract. After incubation at 25°C for 5 min, absorbance at 660 nm was determined (Hitachi U-2000). Authentic trolox (Sigma Chem. Co.) was used as a reference for concentration estimation.

Iron chelating ability was determined according to the method reported by Osawa et al. [16]. Briefly, in a quartz cuvette containing 2.5 ml of 2 μM linoleic acid, 0.05 M of Tris-HCl, 0.15 M of KCl and 0.5% of tween-20 (pH 7.4), 0.5 ml of 0.1 mM FeSO_4 _and 1.5 mM H_2_O_2_, 10 μl of liver extract was added, mixed and incubated at 37°C. At 0 h and 15 h of incubation, the conjugated diene hydroperoxide (CDHP) concentrations were quantified by absorbance determination at 234 nm. Iron chelating ability was expressed by percentage of inhibition of CDHP formation (chelating ability = sample ABS value/control ABS value ×100).

### Histopathological examination of vaginal epithelium

For specimen preparation, the vagina was dissected from each sacrificed rat and subjected to fixation with 10% buffered formalin for 48 h. Epithelial tissues were processed by a tissue processor. Vagina sections (5 μm thickness) were prepared and stained with hematoxylin-eosin. The specimens were then subjected to histopathological examination and photographing. The epithelial cellular linings were classified into two categories, namely, 1–3 and 4–6 layers, for characterization of vaginal degeneration caused by ovariectomy.

### Statistical analyses

Experimental data were analyzed by SAS (statistical analysis system) for variance comparison among the test groups. Tukey's test [19] was subjected to significance analysis according to the following model,



Where Y is a dependent variable, μ represents mean, T is the treatment effect, P is the pen effect and e is the random residual error term. Data are expressed as mean ± SD with a significant difference level at *P *< 0.05.

## Results and discussion

Effects of dietary supplementation with SAI on growth performance of the test rats are shown in Table 2. The average daily gains (ADG) and feed/gain ratios for the ovariectomized (OVX) rats were higher than those of SAI-supplemented rats (*P *< 0.05). Average daily feed intake and energy expenditure for the OVX rats was increased, mostly due to ovariectomy (*P *< 0.05). When comparisons were made among the SAI supplemented groups with the sham group, an insignificant difference on growth performance was observed (*P *< 0.05). This was in agreement with the observation that decrease of estrogen of rats after ovariectomy may result in increase of body weight [20]. Mook et al. [21] addressed that ovariectomy stimulates feed intake of rats. Dietary supplement of estrogen in the diet of rats may result in decrease of feed intake and body weight gain [22].

Effects of diets supplemented with SAI on serum biochemical attributes of the test rats are shown in Table 3. Serum isoflavone concentrations of the SAI supplemented groups were higher than those of the un-supplemented (OVX) group (*P *< 0.05). The OVX group had higher serum cholesterol contents than the sham groups (*P *< 0.05). The lower serum cholesterol levels for the OVX/HSAI group than the OVX groups were observed (*P *< 0.01). However, the serum estradiol levels as affected by SAI supplementation and ovariectomy differed insignificantly among the test groups (*P *< 0.05). Even the SAI supplemented groups had higher estradiol values than the OVX group, the difference was not statistically significant (Table 3).

**Table 2:** Effect of supplementation of soy aglycons of isoflavone on growth performance of Sprague-Dawley rats

Determinations	Rats with different treatments			
	
Items	Sham	OVX	OVX/LSAI	OVX/HSAI
Average daily gain (g)	0.56 ± 0.16^b^	0.83 ± 0.23^a^	0.83 ± 0.20^a^	0.79 ± 0.21^a^
Average daily feed intake (g)	13.74 ± 0.70^b^	17.81 ± 0.98^a^	18.34 ± 1.08^a^	18. 43 ± 1.08^a^
Energy expenditure (kJ/d)	204.0 ± 10.4^b^	264.5 ± 14.6^a^	272.4 ± 16.0^a^	273.7 ± 16.0^a^
Feed/Gain	26.28 ± 3.33^a^	21.24 ± 1.70^b^	22.07 ± 3.80^b^	22.20 ± 3.46^b^

^a, b^Means (± SD, n = 10) in the same row with different superscripts differ significantly (*P *< 0.05). SAI: soy aglycons of isoflavone; Sham: negative control; OVX: overiectomized rats as positive control; OVX/LSAI: overiectomized rats with low level of SAI supplementation; OVX/HSAI: overiectomized rats with high level of SAI supplementation.

**Table 3:** Effect of dietary supplementation of soy aglycons of isoflavone on serum biochemical attributes and lipoprotein profiles of Sprague-Dawley rats

Determinations	Rats with different treatments			
	
Items	Sham	OVX	OVX/LSAI	OVX/HSAI
Estradiol (pg/ml)	65.61 ± 18.30	32.83 ± 17.88	42.18 ± 18.71	50.84 ± 25.48
Isoflavones (daidzein + genistein, μg/ml)	< 0.1^b^	< 0.1^b^	1.28 ± 1.02^a^	1.57 ± 1.20^a^
Cholesterol (mg/dl)	92.75 ± 29.12^a^	129.88 ± 28.11^a^	108.78 ± 33.18^a^	83.75 ± 21.77^b^
Triacylglycerol (mg/dl)	108.12 ± 16.47	115.85 ± 12.52	109.48 ± 13.61	106.08 ± 11.83
HDL (%)	57.28 ± 10.47^ab^	52.26 ± 7.14^b^	53.75 ± 12.42^b^	66.73 ± 10.03^a^
VLDL (%)	11.11 ± 2.98	11.92 ± 2.79	11.10 ± 2.96	9.98 ± 2.61
LDL (%)	31.61 ± 8.67^ab^	35.88 ± 6.14^a^	35.15 ± 9.82^a^	23.29 ± 8.32^b^

^a, b^Means (± SD, n = 10) in the same row with different superscripts differ significantly (*P *< 0.05). SAI: soy aglycons of isoflavone; Sham: negative control; OVX: overiectomized rats as positive control; OVX/LSAI: overiectomized rats with low level of SAI supplementation; OVX/HSAI: overiectomized rats with high level of SAI supplementation.

Soy isoflavones are phytoestrogens with estrogenic activity. Their structures are similar to estrogen and enabling binding competition to the estrogen receptors [23,24]. The binding affinity to estrogen receptor of genistein and daidzein is about 1/100 and 1/1000 of estradiol and known as weak estrogens [25,26]. For humans, Johnson et al. [27] and Messina and Loprinzi [1] suggested that supplement of soy isoflavones for post-menopausal women may reduce risk of incidence of uterus endometrial cancer.

Serum isoflavone (daidzein + genistein) levels of rats for the SAI supplemented groups were higher than those of the sham group (Table 3). It is obvious that serum isoflavone levels were increased by dietary SAI supplementation. This was in agreement with Ishimi et al. [28] who found that serum isoflavone concentrations of mice increase after feeding mice with isoflavones. Doerge et al. [29] also reported that total serum isoflavone levels of aglycons and conjugates of Sprague-Dawley rats fed with soy-free diet were ca. 16 nM while increased up to 5480 nM after orally administered with 34 mg/kg of genistein. For humans, Fanti et al. [4] reported that Japanese consume comparatively more soybean products than people from the Western countries, serum isoflavone levels of the former are comparatively higher than those of the latter.

Effects of dietary supplementation of SAI on lipoprotein profiles of the test rats are shown in Table 3. It was noticed that HDL concentrations of OVX/HSAI were higher than those of OVX group (*P *< 0.05). In further, the LDL concentrations of the OVX/HSAI group were significantly lower than those of OVX group (*P *< 0.05). It is generally agreed that the higher HDL and the lower LDL concentrations are of benefit in chemoprevention of cardiovascular diseases. Some reports in the literature also indicated that soybean isoflavones may prevent coronary heart disease [8] and hyperlipidemia [30]. Integrated consideration of the changes of total cholesterol is also shown in Table 3. It is apparent that dietary supplementation of HSAI has exhibited considerable reduction of serum cholesterol and increase of HDL. Forsythe [31] reported that reduction of serum cholesterol concentration by phytoestrogen may be achieved by increase of the metabolic rate of LDL and VLDL. Nogowski et al. [32] demonstrated that supplementation with 0.1% genistein for 14 days are effective in reduction of serum cholesterol level and lipid synthesis of ovariectomized rats and enhancement of LDL binding to LDL receptor [33]. Reduction of serum cholesterol and lipid biosynthesis may consequently decrease LDL-cholesterol and increase HDL-cholesterol contents [8,34].

Effects of SAI supplementation on liver antioxidative activities are shown in Table 4. The HSAI supplemented groups had higher catalase activity than those of OVX group (*P *< 0.05). The trolox equivalencies were also greater for the HSAI supplemented groups than those of OVX group (*P *< 0.01). Conversely, the TBARS values were lower for the SAI supplemented groups than those of OVX group (*P *< 0.01). Iron chelating ability was greater for the SAI supplemented groups than those of the OVX group (*P *< 0.05). Other test items including GSH Px activity, POV and CDHP contents did not differ among the test groups (*P *< 0.05). Tikkanen et al. [35] reported that genistein is able to depress hydroperoxide generation; and increase antioxidative enzyme activities of SOD, catalase, GSH Px and GSH reductase [36]. Bazzoli et al. [37] reported that increase of serum total antioxidant status (trolox equivalency) is enhanced for young women after receiving soybean (40 g protein/d) for 4 weeks. Kapiotis et al. [38] also reported that genistein and daidzein may decrease cellular TBARS level. Genistein inhibits conjugate diene hydroperoxide formation but enhances trolox equivalency, and iron chelating and OH^- ^radical scavenging activities. Soy isoflavones contain three OH groups and offer H atoms to quench free radicals by a chain reaction [5]. In this study, enhanced iron chelating ability achieved by SAI supplementation was also observed (Table 4). This was in agreement with Lee et al. [7] who reported that soy aglycons of isoflavone possess ferric reducing-antioxidant power. Kerry and Abbey [6] demonstrated in an *in vitro *study that genistein (5–200 μmol/L) inhibits copper and peroxyl radical-mediated LDL oxidation.

**Figure 1:**
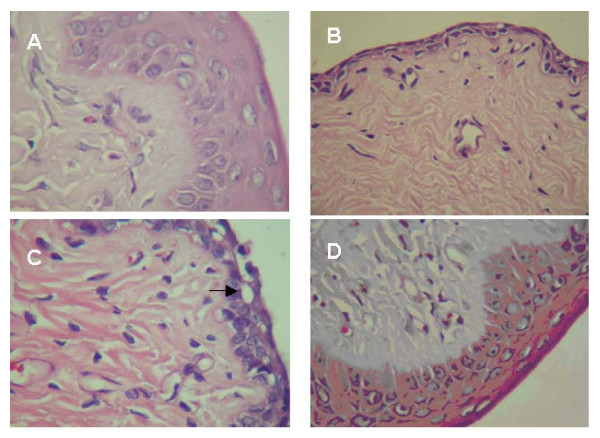
**Histopathological examination of the vaginal epithelia as affected by diet supplementation with soy aglycons of isoflavone**. A: sham rats, 4–6 layers of the normal epithelial cellular linings; B: OVX rats, 1–3 layers of the epithelial cellular linings; C: OVX rats, empty vacuole (as indicated by an arrow) or loosen cell debris; and D: OVX/HSAI rats, maintaining 4–6 layers of the epithelial cellular linings.

Effects of SAI supplementation on protection of the vaginal epithelium from degeneration due to ovariectomy are shown in Figure 1. Normally, 4–6 layers of the epithelial cellular linings were observed for the sham rats (Figure 1A). In comparison, degeneration of the epithelial cells and some empty vacuoles and loosen cell debris have been noticed for the OVX rats (Figures 1B and 1C). For rats of the SAI supplemented groups, degeneration of the epithelial cellular linings have been less pronounced (Figure 1D). As examined based on the rat ratios bearing 4–6 layers of epithelial cellular linings for sham, OVX, OVX/LSAI and OVX/HSAI groups were 100, 28.6, 30.0 and 42.9%, respectively. Thus, dietary supplement of soy isoflavones in improvement of vaginal epithelium from degeneration due to ovariectomy-caused shortage of estrogen is obvious. This was in agreement with Malaivijitnond et al. [39] who reported that supplementation of genistein (0.25–2.5 mg/kg BW) induces vaginal cornification. Kim et al. [40] also reported that vaginal blood flow of rats decrease by ovariectomy while increase by estradiol supplementation.

**Table 4:** Effect of supplementation of soy aglycons of isoflavone on antioxidative capacities of rat liver extracts

Determinations	Rats with different treatments			
	
Items	Sham	OVX	OVX/LSAI	OVX/HSAI
SOD (Unit)	21.81 ± 9.44	14.84 ± 8.95	25.55 ± 11.62	18.44 ± 9.40
Catalase (Unit)	6.80 ± 1.60^b^	5.46 ± 2.36^b^	8.14 ± 1.59^ab^	8.47 ± 0.87^a^
GSH Px (Unit)	0.30 ± 0.12	0.53 ± 0.32	0.38 ± 0.35	0.54 ± 0.44
Trolox equivalent (millimole/l)	187.25 ± 46.60^ab^	140.00 ± 20.51^b^	181.2 ± 24.00^ab^	213.14 ± 21.13^a^
TBARS (nanomole MDA/ml)	82.84 ± 5.68^b^	94.70 ± 1.62^a^	82.76 ± 3.73^b^	86.56 ± 4.92^b^
Chelating iron (%)	74.60 ± 6.04^b^	77.75 ± 5.62^ab^	82.32 ± 2.25^a^	81.01 ± 1.94^a^
POV (%)	94.13 ± 1.15	94.20 ± 1.00	93.29 ± 1.47	94.30 ± 0.97
Inhibition of conjugated diene peroxide formation (%)	24.30 ± 6.10	23.88 ± 5.03	22.61 ± 6.37	22.37 ± 8.40

^a, b^Means (± SD, n = 10) in the same row with different superscripts differ significantly (*P *< 0.05). SAI: soy aglycons of isoflavone; Sham: negative control; OVX: overiectomized rats as positive control; OVX/LSAI: overiectomized rats with low level of SAI supplementation; OVX/HSAI: overiectomized rats with high level of SAI supplementation; SOD: superoxide dismutase; GSH Px: glutathione peroxidase; TBARS: thiobarbituric acid-reactive substance; POV: peroxide value.

## Conclusion

Health benefits evidenced by detection of higher serum isoflavone concentrations, significantly lower serum cholesterol and LDL contents, higher HDL contents, increased iron chelating ability, lower liver TBARS (thiobarbituric acid-reactive substance) contents, enhanced liver catalase and total antioxidative (as trolox equivalency) activities, and protection of the epithelial cellular linings of vagina of the OVX rats have been achieved by dietary supplementation with soy aglycons of isoflavone. The observations that dietary SAI supplementation in performance of estrogenic effectiveness in improvement of serum biochemical attributes, enhancement of liver antioxidative capacities and protection of vaginal epithelium are of importance from the viewpoint of healthcare and development of dietary supplements. Apparently, diets supplemented with soy aglycons of isoflavone have conferred health benefits to the OVX rats. This further supports the effectiveness that menopausal-related syndromes could be prevented or improved by dietary supplementation with soy aglycons of isoflavone.

## Authors' contributions

TFL and YLH carried out most experiments and drafted the manuscript. DYL carried out histopathological examinations. RYC conceived of this study, and participated its design and coordination. All authors read and approved the final manuscript.
